# Impact of supra-aortic vessel false lumen communications on aortic remodeling: Analysis from the PERSEVERE trial

**DOI:** 10.1016/j.xjse.2025.100081

**Published:** 2025-10-08

**Authors:** Kyle Eudailey, Sabin Bozso, Shinichi Fukuhara, Fernando Fleischman, Ibrahim Sultan, William Brinkman, Hiroo Takayama, Arminder Jassar, George Arnaoutakis, Michael C. Moon, Wilson Y. Szeto

**Affiliations:** a Division of Cardiac Surgery, Department of Surgery, University of Alabama at Birmingham, Birmingham, Ala; b Department of Cardiac Surgery, University of Michigan, Ann Arbor, Mich; c USC Cardiac and Vascular Institute, University of Southern California, Los Angeles, Calif; d Division of Cardiac Surgery, University of Pittsburgh Medical Center, Pittsburgh, Pa; e Cardiac Surgery Specialists, Thoracic Aortic Clinic, The Heart Hospital Baylor Plano, Plano, Tex; f Division of Cardiac, Thoracic & Vascular Surgery, Columbia University Irving Medical Center/New York Presbyterian Hospital, New York, NY; g Division of Cardiac Surgery, Department of Surgery, Massachusetts General Hospital, Boston, Mass; h Division of Cardiovascular and Thoracic Surgery, University of Texas Austin, Austin, Tex; i Division of Cardiac Surgery, Department of Surgery, University of Alberta, Edmonton, Alberta, Canada; j Division of Cardiovascular Surgery, University of Pennsylvania, Philadelphia, Pa

**Keywords:** aorta, acute aortic dissection, AMDS hybrid prosthesis aortic remodeling, hemiarch, malperfusion

## Abstract

**Objective:**

The PERSEVERE (ProspEctive, Single ARm, Multi-center Clinical InveStigation to EValuatE the Safety and Effectiveness of AMDS in the TREatment of Acute DeBakey Type I Dissection) Trial is a US investigational device exemption study designed to evaluate the Ascyrus Medical Dissection hybrid prosthesis in the open repair of patients with acute DeBakey type I dissection with preoperative malperfusion. We sought to determine the impact of false lumen (FL) communications within the supra-aortic vessels (SAV) on aortic remodeling by analyzing total aortic diameter (TAD), FL diameter, and true lumen (TL) diameter.

**Methods:**

Ninety-three patients were enrolled from July 2022 to November 2023. Anatomic measurements were made by a core laboratory using computed tomography angiogram (CTA). For this study, analysis was limited to zones 2, 3, and 4. TAD was assessed on first postoperative CTA and at 1-year postoperatively, and growth was defined as >1.0 cm. In addition, the percent of patients with FL diameter larger than TL diameter were compared on the basis of the presence or absence of any FL communications in the SAV.

**Results:**

Fifty-five patients had a preoperative, first postoperative CTA, and 1-year CTA scan and were included in the analysis. SAV FL communications were present on preoperative computed tomography scan in 35% (19/55) and absent in 65% (36/55). In those with SAV FL communications, TAD growth occurred in 0%, 5.3%, and 21.1% in zones 2, 3, and 4, respectively. In those without SAV FL communications, TAD growth occurred in 2.8%, 2.8%, and 8.3% in zones 2, 3 and 4, respectively; the difference did not reach statistical significance. The FL was larger than TL in significantly more patients in zones 3 and 4 in those with compared with those without SAV FL communications (zone 3, 53% vs 18%; zone 4, 58% vs 18%; *P* < .05).

**Conclusions:**

The presence of preoperative SAV FL communications has a significant impact on aortic remodeling at 1-year postoperatively. The presence of these communications is associated with a trend toward negative remodeling in zones 2, 3, and 4, in addition to a significantly larger FL compared with TL diameter, likely representing ongoing FL pressurization. Techniques to better identify SAV FL communications preoperatively may lead to a more tailored approach to operative management and more targeted postoperative surveillance in these patients.

**Figure:**
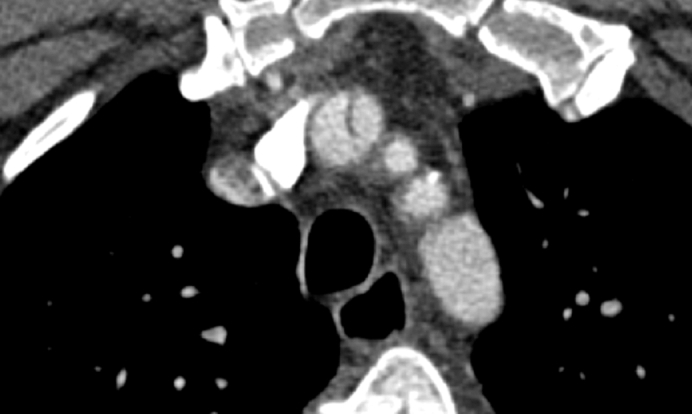
Innominate artery false lumen communication as demonstrated on preoperative CT.

**Central Message**

Techniques to better identify supra-aortic vessel false lumen communications may lead to a more tailored approach to operative management including use of the AMDS.

**Perspective**

Supra-aortic vessel false lumen communications lead to adverse aortic remodeling in the downstream aorta, specifically zones 3 and 4. Attention should be paid to preoperative imaging and intraoperative examination to identify these communications. Modification of techniques may be necessary to help to reduce the impact of these communications.

Acute DeBakey type I aortic dissection (ADTI) is a clinical emergency requiring surgical intervention to prevent mortality and reduce morbidity.^1^^,^^2^ The Ascyrus Medical Dissection Stent (AMDS) Hybrid Prosthesis (Artivion) is a novel, self-expanding, braided nitinol stent designed to treat ADTI, in conjunction with a surgical graft in a proximal surgical repair. AMDS has previously undergone investigation in the Dissected Aorta Repair Through Stent (DARTS) Implantation trial^3^, ^4^, ^5^ and is now under investigation in the PERSEVERE (ProspEctive, Single ARm, Multi-center Clinical InveStigation to EValuatE the Safety and Effectiveness of AMDS in the TREatment of Acute DeBakey Type I Dissection) investigational device exemption study in the United States. The investigational device exemption study restricted enrollment to a high-risk population of patients with preoperative malperfusion.^6^

The AMDS has been associated with positive aortic remodeling in the treated segment, including the aortic arch and proximal descending aorta, in both the DARTS and PERSEVERE studies. However, in some patients in the DARTS study, preoperative false lumen (FL) communications in the supra-aortic vessels (SAVs) have been predictive of adverse aortic remodeling beyond 1-year postprocedure.^3^^,^^7^^,^^6^^,^^8^^,^^9^ Because of the limited sample size in the DARTS study, we sought to determine the early impact of preoperative FL communications within the SAVs on aortic remodeling through 1 year by analyzing changes in total aortic diameter (TAD), true lumen (TL) diameter, and FL diameter in patients treated in the PERSEVERE study. We hypothesized that the presence of SAV FL communications would be associated with more negative remodeling in the downstream aorta.

## Methods

### Study Design and End Points

The PERSEVERE study is a single-arm, nonblinded trial with 26 sites activated in the study and 93 patients enrolled between July 2022 and November 2023. Each site was approved by the overseeing institutional review board (central institutional review board reference number 20216348; initial approval on December 17, 2021). Between July 2022 and November 2023, 118 patients were prospectively consented for the study. In 25 of these 118 cases, a legally authorized representative was required because the patient was unable to provide written consent. The details of this trial have been reported previously.^6^ Eligible adults (18-80 years old) with ADTI and clinical and/or radiographic malperfusion were considered for enrollment. Patients were excluded for the following criteria: primary entry tear extends into the aortic arch or a total arch replacement is required, life expectancy <2 years (due to other medical condition), pregnant or breastfeeding, unwilling to accept blood transfusions, present with coronary malperfusion, were in circulatory shock or extreme hemodynamic compromise, and had a connective tissue disorder.^6^

Study end points included the incidence of major adverse events, as well as radiographic assessments of aortic remodeling on the basis of independent imaging Core Laboratory evaluation of computed tomography angiogram (CTA) data using centerline measurements.^6^ The primary objectives of this study are evaluating changes in TAD, TL diameter, and FL diameter at 1 year.

### Statistical Analysis

The aortic remodeling results presented are based on an ad hoc analysis of prospectively collected data. Preoperative communications in the SAVs were selected as a variable of interest because of the results of the DARTS study analysis.^7^ Patients were grouped on the basis of existence or absence of ≥1 FL communication in the SAVs, including the innominate artery, left common carotid artery, or the left subclavian artery. Negative aortic remodeling was considered as the following within 1 year: TAD growth >1.0 cm, TL reduction >5.0 mm, and FL expansion >5.0 mm. TAD baseline was the 1st postoperative CTA and TL and FL baseline diameter was from preprocedure CTAs. Reoperative risks after dissection repair can be much greater than index operations and therefore after extensive discussions among the original PERSEVERE trial investigators, a greater threshold of 1 cm in 12 months was chosen as the most clinically relevant indication of a potential need for reintervention. All aortic measurements are provided using descriptive measures. Measurements were taken in zones 2, 3, and 4 for each comparison. Continuous variables are described using median and interquartile range. Categorical variables were described using n and % of the total. Change in TAD, TL, and FL diameters were compared between groups ± SAV communications using a Fisher exact test. The percent of patients with an FL diameter greater than a TL diameter at 1 year was compared between groups ± SAV communications using a Pearson χ^2^ test. All analyses were performed in SAS (version 9.4).

## Results

### Baseline Demographics

Of the 93 patients enrolled, there were 55 patients who had a preoperative CTA, an early postoperative CTA, and a 1-year CTA available for the analysis. Of the 55 patients, 19 (35%) patients had preoperative FL communications in the SAVs and 36 (65%) did not. Baseline demographics for the included study population are reported in Table 1. The mean age of the study population was 58.8 years. Forty-six (83.6%) patients were male, 26 (47.3%) patients had hypertension, 20 (36.3%) patients had chronic kidney disease, and 18 (32.7%) patients had dyslipidemia.

**Table 1:** Baseline demographics of the included study population

Variable	With SAV FL communication (n = 19)	Without SAV FL communication (n = 36)
Age, y	59.0 ± 9.6	58.7 ± 9.1
BMI, kg/m^2^	29.7 ± 6.4	29.8 ± 6.2
Male	84% (n = 16)	83% (n = 30)
White	47% (n = 9)	67% (n = 24)
Black	37% (n = 7)	11% (n = 4)
Hispanic	16% (n = 3)	11% (n = 4)
Arterial hypertension	47.3% (n = 9)	47.2% (n = 17)
Hyperlipidemia	42.1% (n = 8)	38.9% (n = 14)
CKD	36.8% (n = 7)	38.2% (n = 13)
Type II diabetes	21.1% (n = 4)	8.3% (n = 3)
Cancer	10.5% (n = 2)	11.1% (n = 4)
History of tobacco use	47.4% (n = 9)	47.2% (n = 17)
Illicit drug use	0% (n = 0)	19.4% (n = 7)

*SAV*, Supra-aortic vessel; *FL*, false lumen; *BMI*, body mass index; *CKD*, chronic kidney disease.

#### TAD change

**Figure 1:**
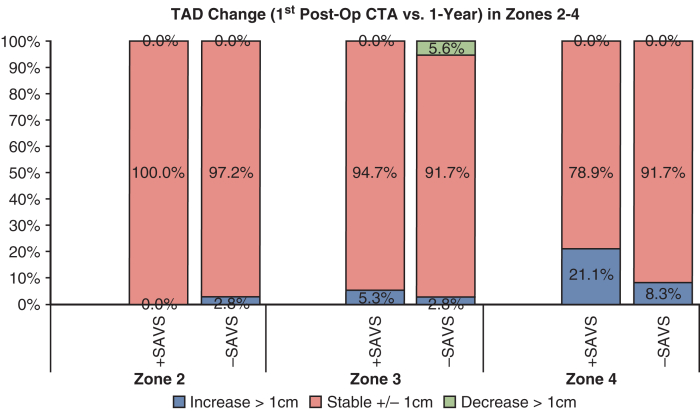
Bar graph demonstrating changes in total aortic diameter from 1st postoperative CTA to 1 year CTA measurements in zones 2, 3, and 4 for patients with and without supra-aortic vessel false lumen communications. *CTA*, Computed tomography angiogram; *TAD*, total aortic diameter.

**Figure 2:**
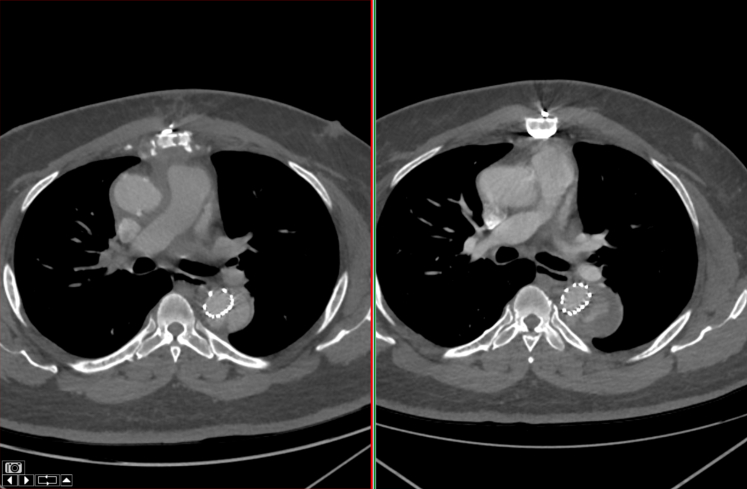
Negative aortic remodeling in presence of supra-aortic vessel false lumen communications at 30-day and at 1-year follow-up.

Overall, TAD growth was observed in a minority of patients (≤21%) in zones 2, 3, and 4 (Table E1). In patients with SAV FL communications, TAD growth occurred in 0% (0/19), 5.3% (1/19), and 21.1% (4/19) in zones 2, 3, and 4, respectively (Figure 1). In those without SAV FL communications, TAD growth occurred in 2.8% (1/36), 2.8% (1/36), and 8.3% (3/36) in zones 2, 3, and 4, respectively. There was no significant difference between groups. Four of the 19 patients had more than 1 supra-aortic FL communication. All 4 patients experienced growth >1 cm at 1 year in zones 3 and 4 and 3 of the 4 patients in zone 2 (Figure 2).

#### True lumen diameter change

**Figure 3:**
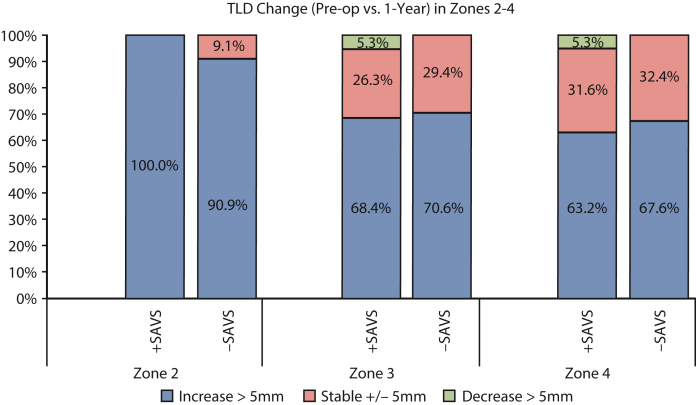
Bar graph demonstrating changes in true lumen diameter (TLD) from preoperative to postoperative CTA measurements in zones 2, 3, and 4 for patients with and without supra-aortic vessel false lumen communications. *CTA*, Computed tomography angiogram.

Overall, TL diameter reduction was observed in a minority of patients (≤5.3%) in zones 2, 3, and 4 (Table E2). In patients with SAV FL communications, TL size decreased in 0% (0/19), 5.3% (1/19), and 5.3% (1/19) in zones 2, 3 and 4, respectively. In those without SAV FL communications, TL size decreased in 0% (0/36) in zones 2, 3, and 4 (Figure 3). There was no significant difference between groups.

### FL Thrombosis and Diameter Change

**Figure 4:**
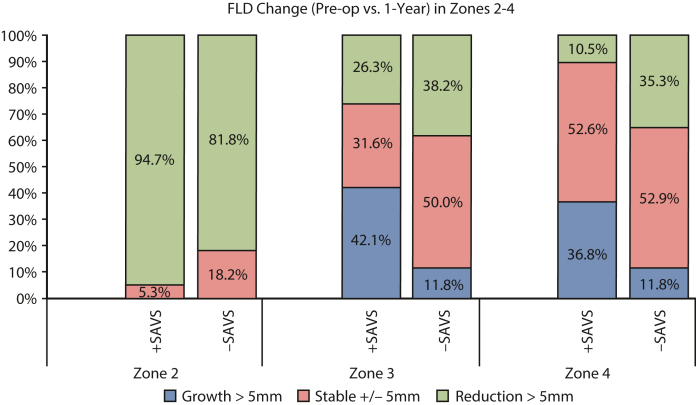
Bar graph demonstrating changes in false lumen diameter (FLD) from preoperative to postoperative CTA measurements in zones 2, 3, and 4 for patients with and without supra-aortic vessel false lumen communications. *CTA*, Computed tomography angiogram.

There was no difference in FL thrombosis status between groups at 1 year (Table E3). In patients with SAV FL communications, there was complete or partial FL thrombosis in 91.2% in zone 2, 87.9% in zone 3, and 87.9% in zone 4. In patients without SAV FL communications, there was complete or partial FL thrombosis in 94.8% in zone 2, 89.5% in zone 3, and 100% in zone 4. FL reduction >5 mm was observed in a majority of patients in Zone 2 in patients with SAV FL communications (94.7% [18/19]) and without SAV FL communications (83.3% [30/36]) (Figure 4). In patients with SAV FL communications, FL diameter increased in 42.1% and 36.8% in zones 3 and 4, respectively. In those without SAV FL communications, FL diameter increased in 11.8% in zones 3 and 4 (Figure 4). There was no significant difference between groups. At 1 year, the FL > TL ratio was the same in the +SAV and –SAV groups (0%) in zone 2. At 1 year, the FL > TL ratio was significantly greater in the +SAV group compared with the –SAV group in zone 3 (53% vs 18%) and zone 4 (58% vs 18%) (*P* < .05).

## Discussion

**Figure 5:**
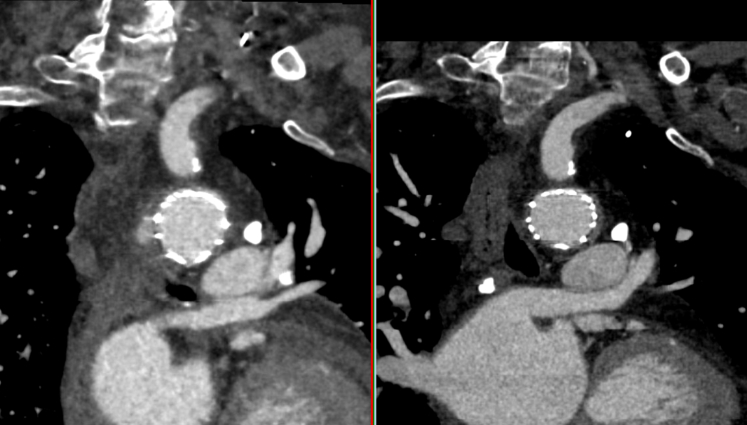
Positive aortic remodeling in absence of supra-aortic vessel false lumen communications at 30-day and at 1-year follow-up.

In this study, we conducted an ad hoc analysis of the PERSEVERE trial data investigating predictors of aortic remodeling and identified several key points. The presence of preoperative SAV FL communications had an impact on aortic remodeling at 1-year postoperatively. The presence of these communications is associated with a trend toward negative remodeling in a subset of patients in zones 3 and 4, in addition to a significantly larger FL compared with TL diameter, likely representing ongoing FL pressurization (Figures 2 and 5).

Previous studies have sought to identify preoperative predictors of adverse aortic remodeling after ADTI repair with the AMDS Hybrid Prosthesis. A previous study by EL-Andari and colleagues^7^ investigated predictors of adverse aortic remodeling in the DARTS cohort with 3 years of follow-up. Their study found that FL communications in the SAVs was associated with significant aortic growth at zone 3 and that FL communications in the visceral vessels was associated with adverse remodeling in zones 3, 6, and 9. The main limitation of this previous study was sample size with 29 of the original 46 patients having CT scans at 3 years, which this present study has helped to address and was able to identify similar findings.

The identification of preoperative predictors of adverse aortic remodeling may help to guide initial interventions for patients with ADTI. In this cohort, the presence of preoperative SAV FL communications had an impact on aortic remodeling at 1 year. This suggests that the FL perfusion in these branch vessels results in retrograde perfusion of the aortic FL and is associated with aortic degeneration after repair. The AMDS does not address these branch vessel communications as a total arch replacement would. It is, therefore, imperative that these FL communications are identified on preoperative imaging to guide initial management. Techniques to better identify SAV FL communications are needed, as well as dedicated time and attention to identifying these communications preoperatively, which may lead to a more tailored approach to operative management and more targeted postoperative surveillance in these patients.

### Limitations

One limitation of this study is the limited follow-up time of 1 year. Adverse aortic remodeling will continue to evolve over time, and additional follow-up will help to discern which patients will have clinically significant aortic remodeling requiring surgical or endovascular reinterventions. Further, only 55 of the 93 patients in PERSEVERE (59%) had a preoperative, 1st postoperative, and 1-year CT scan and were included in the study. Since the main outcomes of this study were the impact of SAV FL communication on remodeling, those time points were all necessary for inclusion, despite not all patients having a 1st post-operative CT. Finally, the overall sample size is small, and the study is subject to selection bias.

## Conclusions

The presence of preoperative SAV FL communications is associated with adverse aortic remodeling in the aortic arch and proximal descending thoracic aorta after ADTI repair with the AMDS. Close attention should be given to the SAVs at the time of review of the preoperative CT scan to identify FL communications that may predict adverse aortic remodeling. Longer-term follow-up of the patients in the PERSEVERE trial will be valuable in identifying whether the short-term adverse aortic remodeling identified in this study results in clinically significant outcomes, such as required reintervention.

### Webcast

You can watch a Webcast of this AATS meeting presentation by going to: https://www.aats.org/resources/impact-of-supra-aortic-vessel--10309.

**Figure:**
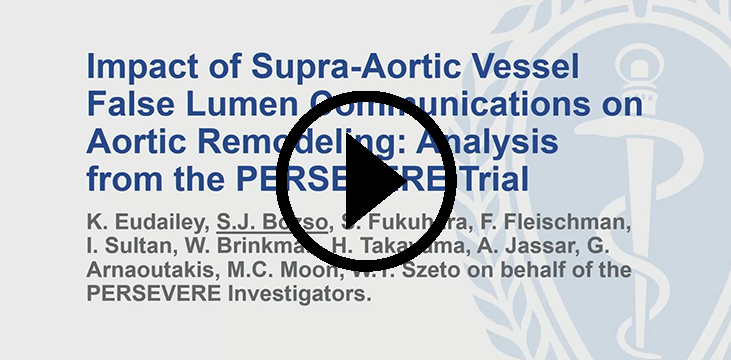

### Audio

You can listen to the discussion audio of this article by going to the supplementary material section below.

## Conflict of Interest Statement

The study was sponsored by Artivion, Inc. All authors are PERSEVERE Steering Committee members and/or investigators on the study.

The *Journal* policy requires editors and reviewers to disclose conflicts of interest and to decline handling or reviewing manuscripts for which they may have a conflict of interest. The editors and reviewers of this article have no conflicts of interest.

## Figures and Tables

**Table E1:** TAD change (first postoperative CTA vs 1-year CTA), zones 2-4

Zone	SAV (n = 19)	No SAV (n = 36)
Zone 2, mm		
Mean ± SD	2.0 ± 2.7	1.9 ± 3.4
Median	1.9	1.6
Min	−1.8	−5.4
Max	7.5	13.3
Zone 3, mm		
Mean ± SD	4.2 ± 4.7	1.9 ± 5.6
Median	6.5	2.6
Min	−5.4	−12.6
Max	12.1	14.5
Zone 4, mm		
Mean ± SD	6.7 ± 4.8	2.7 ± 5.0
Median	5.3	2.8
Min	−0.8	−6.4
Max	21.5	12.8

*SD*, Standard deviation.

**Table E2:** TLD change (Pre-Op CTA vs 1-Year) zones 2-4

Variable	SAV (n =19)	No SAV (n = 36)
Zone 2		
Mean ± SD	14.3 ± 4.1	12.7 ± 5.4
Median	13.9	12.9
Min	5.2	1.2
Max	23.5	21.3
Zone 3		
Mean ± SD	7.1 ± 5.7	8.0 ± 5.3
Median	6.7	8.1
Min	−5.5	−3.7
Max	15.5	18.5
Zone 4		
Mean ± SD	7.4 ± 5.9	8.1 ± 5.5
Median	8.2	7.7
Min	−7.5	−2.9
Max	15.8	21.1

*SAV*, Supra-aortic vessel; *SD*, standard deviation.

**Table E3:** FLD change (preoperative CTA vs 1-year CTA) for zones 2-4

Variable	SAV (n = 19)	No SAV (n = 36)
Zone 2		
Mean ± SD	−10.6 ± 4.3	−10.3 ± 7.1
Median	−11.3	−9.7
Min	−17.3	−25.9
Max	0.2	2.7
Zone 3		
Mean ± SD	1.1 ± 9.3	−3.5 ± 7.9
Median	1.0	−1.4
Min	−17.0	−19.4
Max	17.4	10.2
Zone 4		
Mean ± SD	3.7 ± 8.8	−2.1 ± 7.3
Median	2.4	−0.9
Min	−13.9	−19.2
Max	22.7	19.6

*SAV*, Supra-aortic vessel; *SD*, standard deviation.

## Supplementary Data

### Audio

Audio 1 This is an audio recording of the presentation of the associated abstract and discussion. The abstract presentation ends at 5:53 and the discussion begins at 5:57.
